# Brief Admission by Self‐Referral: A 4‐Year Follow‐Up on Utilisation Patterns and Experiences

**DOI:** 10.1111/inm.70091

**Published:** 2025-07-09

**Authors:** Daiva Daukantaitė, Rose‐Marie Lindkvist, Reid Lantto, Sofie Westling

**Affiliations:** ^1^ Department of Psychology Lund University Lund Sweden; ^2^ Department of Psychiatry Skåne University Hospital Lund Sweden; ^3^ Psychiatry Department of Clinical Sciences Malmö, Lund University Lund Sweden; ^4^ Department of Child and Adolescent Psychiatry Skåne University Hospital Helsingborg Sweden

**Keywords:** borderline personality disorder, brief admission, long‐term follow‐up, self‐harm, self‐referral

## Abstract

Brief Admission by self‐referral (BA) is a crisis intervention for individuals with recurrent self‐harm and suicidal ideation. While short‐term effects are documented, long‐term utilisation patterns remain unclear. This study examines BA usage over 4 years, identifies distinct utilisation profiles, and qualitatively explores participants' experiences with BA over time. Participants were 62 individuals from a prior randomised controlled trial who provided informed consent for follow‐up. Using a mixed‐methods convergent parallel design, quantitative and qualitative data were collected simultaneously, analysed separately, and integrated during the discussion. BA utilisation and profiles were examined quantitatively, while qualitative content analysis was applied to open‐ended responses. When analysing mean levels across the entire sample, BA usage initially averaged 8 days per 6‐month period but gradually declined over 4 years to 3–4 days. However, cluster analysis revealed distinct BA usage trajectories across three utilisation profiles: Cluster 1 (*n* = 40) exhibited consistently low BA usage, Cluster 2 (*n* = 14) showed a gradual decline following an initial phase of engagement, and Cluster 3 (*n* = 8) maintained high and sustained BA usage throughout the 4‐year period, reporting greater impairments but strong satisfaction with BA. These individuals valued BA for its structured support, autonomy, and sense of security. BA appears to serve as both a form of self‐care and a gateway to broader psychiatric support, particularly for those with greater functional impairments. To optimise its long‐term effectiveness, structural barriers, access inconsistencies, and stigma must be addressed through better integration into psychiatric services.

Originating in the Netherlands in the 1990s as ‘bed‐on‐prescription’ (van Veldhuizen et al. 1988), several initiatives have emerged over the last decade in the Nordic countries, allowing individuals with psychiatric disorders to admit themselves to brief psychiatric inpatient care (Eckerström et al. 2019; Sigrúnarson et al. 2017; Thomsen et al. 2018; Westling et al. 2019), further expanding to the UK (Psychiatry UK, n.d.), where self‐referral inpatient programmes are emerging. The models have been assigned various descriptive names, including Self‐Referral to Inpatient Treatment (SRIT; Sigrúnarson et al. 2017), Patient‐Controlled Admission (PCA; Thomsen et al. 2018), Brief Admission by self‐referral (BA; Westling et al. 2019), and Patient‐Initiated Brief Admission (PIBA; Eckerström et al. 2022). Although these models do not differ significantly to justify distinct names, there is a lack of international consensus on terminology. Based on the literature, BA is the most used term and was also the one used in the randomised controlled trial (RCT) upon which this study is based (Westling et al. 2019). Therefore, we will use the term BA throughout this article when referring to more than one of the models.

BA represents a patient‐centred psychiatric care model that allows individuals with severe mental health conditions to initiate short voluntary hospital admissions without requiring an external referral (Liljedahl et al. 2017). The intended target groups vary across settings but may include individuals with suicidal ideation who self‐harm, individuals with psychosis, or individuals with other forms of severe mental illness, at times requiring psychiatric inpatient services. Generally aimed at promoting autonomy, preventing deterioration, and addressing needs as they arise, these BA models have been welcomed among users, relatives, and clinicians alike (e.g., Olsø et al. 2016; Eckerström et al. 2019; Lindgren et al. 2024; Eckerström et al. 2020; Lindkvist, Westling, Liljedahl, and Landgren 2021; Lindkvist, Westling, Eberhard, et al. 2021; Moljord et al. 2021; Hultsjö, Rosenlund, et al. 2023). Most models involve the negotiation of an agreement that grants access to self‐referral (Maconick et al. 2023). This agreement often stipulates pre‐specified conditions for how to use BA, generally aimed at enhancing autonomy. It also includes individualised parts on goals and needs as well as the type of help that can be expected and provided in the BA setting (Liljedahl et al. 2017). BA is provided as an addition to regular treatment, and individuals with access to BA also have the option to seek emergency care as needed (Moljord et al. 2021; Lantto et al. 2023).

The initial promising results of BA, which suggested a reduction in health care utilisation and involuntary care, were hampered by the lack of control groups (Strand and von Hausswolff‐Juhlin 2015). However, subsequent randomised trials and a large‐scale register study incorporating control groups have not demonstrated significant differences in inpatient utilisation between individuals with and without access to BA (Sigrúnarson et al. 2017; Thomsen et al. 2018; Westling et al. 2019). Findings on the economic impact of BA remain inconclusive. While cost analyses have not consistently shown significant differences in healthcare costs between BA users and controls (Lindkvist, Steen Carlsson, et al. 2024; Moljord et al. 2021; Paaske et al. 2021), some studies indicate meaningful benefits in resource allocation (Strand et al. 2021) and for participants' mental health (Lindkvist, Steen Carlsson, et al. 2024). For instance, intra‐individual comparisons suggest improvements in health‐related quality of life from intake to discharge (Eckerström et al. 2022) and enhanced daily functioning (Westling et al. 2019). Furthermore, a health economic analysis based on a randomised controlled trial by Westling et al. (2019) reported a significant increase in quality‐adjusted life years (QALYs) for BA participants over 1 year—equivalent to approximately one additional month of perfect health (Lindkvist, Steen Carlsson, et al. 2024).

Alongside these more modest positive effects observed in quantitative studies, qualitative research has provided deeper insight into users' and relatives' lived experiences, emphasising benefits that extend beyond measurable clinical outcomes. Users overwhelmingly describe BA as a safe and structured respite that fosters autonomy, self‐care, and trust in the healthcare system (Olsø et al. 2016; Helleman et al. 2018; Lindkvist, Westling, Liljedahl, and Landgren 2021; Lindkvist, Westling, Eberhard, et al. 2021; Eckerström et al. 2020; Enoksson et al. 2022). Relatives, who often endure significant stress and fear as their loved ones are at risk of harm, view BA as vital support that improves relationships and daily life (Hultsjö, Appelfeldt, et al. 2023; Lantto et al. 2023; Lindkvist, Eckerström, et al. 2024). Clinicians also report positive experiences, citing increased sense of safety and improved inpatient–outpatient continuity (Westling et al. 2019; Lindgren et al. 2024). However, implementation challenges remain. Users and relatives report clinician non‐adherence to BA principles and limited bed availability as key issues (Eckerström et al. 2020; Helleman et al. 2018; Lindkvist, Westling, Liljedahl, and Landgren 2021; Lindkvist, Westling, Eberhard, et al. 2021; Lindkvist, Eckerström, et al. 2024). Additionally, negative clinician attitudes and frequent rejections due to bed shortages can leave relatives feeling helpless and erode users' trust in the system, discouraging timely help‐seeking (Lantto et al. 2023; Lindkvist, Eckerström, et al. 2024).

Despite its promising potential of reducing the length of individual hospital stays (Eckerström et al. 2024), the long‐term impact of BA remains unclear. Research has yet to determine how BA utilisation evolves over time, whether distinct usage patterns exist, and if so, how they may relate to health and well‐being outcomes. To address these gaps, this study aims to (1) examine BA utilisation over a 4‐year follow‐up period to provide the first long‐term perspective on usage trends, (2) identify distinct BA utilisation profiles and determine whether these differ in key demographic, health and functional variables at follow‐up, and (3) qualitatively interpret BA utilisation by analysing participants' open‐ended accounts of their reasons for and experiences with using BA over at least 4 years of access.

## 1 Materials and Methods

### 1.1 Design

This study employs a mixed‐methods approach using a convergent parallel design, in which quantitative and qualitative data were collected simultaneously, analysed independently, and then integrated during the discussion to interpret the findings collectively (Creswell and Plano Clark 2017).

### 1.2 Participants and Setting

The RCT, registered at ClinicalTrials.gov (NCT02985047), was conducted between 2015 and 2018 and is described in detail in Westling et al. (2019). This 4‐year follow‐up study was conducted from 2018 to 2022 and focuses on long‐term outcomes.

All 125 individuals who participated in the original RCT were considered eligible for the follow‐up study. At the time of inclusion, each participant had a documented history of self‐harm and/or recurrent suicidal behaviour and met at least three diagnostic criteria for borderline personality disorder (BPD). In addition to self‐harm and suicidal behaviour—reported by all participants—the most commonly endorsed additional criteria were emotional instability characterised by predominantly negative affect and a persistent sense of emptiness, both reported by over 80% of the sample. Participants were recruited from four psychiatric inpatient clinics across Skåne, a region in southern Sweden with a population of approximately 1.4 million. The original trial evaluated the effects of BA, specifically targeting individuals at high risk for suicide. During the RCT, all participants continued receiving care through their respective psychiatric outpatient clinics and were randomly assigned to receive either BA in addition to treatment as usual (TAU) or TAU alone for a duration of 12 months.

Of the 125 eligible individuals, 81 provided informed consent to participate in the follow‐up and constituted the initial analytical sample. The remaining 44 were either unreachable, declined to participate, or were excluded for various reasons (e.g., suicide, relocation, or sentencing to forensic psychiatric care). During the 4‐year follow‐up, 19 of the 81 participants discontinued participation due to suicide or complications from self‐harm (*n* = 6), relocation outside the catchment area (*n* = 5), withdrawal of consent (*n* = 5), death from other causes (*n* = 2), or sentencing to forensic psychiatric care (*n* = 1). The final analytical sample consisted of 62 participants who completed the full 4‐year follow‐up.

### 1.3 Implementation of BA

BA was administered according to a structured manual to ensure consistency (Liljedahl et al. 2017) with strategies to enhance treatment fidelity (Westling et al. 2019). Each participant negotiated an individualised contract outlining the objectives, needs, and conditions for self‐admission. When participants got BA access, they could self‐admit for up to three nights, at most three times per month. The decision to use BA was made by the participants themselves. During their stay, participants were responsible for bringing their medication and maintaining their own safety. They had the option to attend two supportive meetings daily with nurses' aides at the unit. However, they did not have access to physicians, psychologists, or other healthcare professionals at the unit during their stay. Such contacts continued in outpatient care. Registered nurses were only occasionally involved and, when present, took on the same supportive role as the nurses' aides. Participants could leave the unit during the day for outpatient care or other personal activities. Instances of self‐harm resulted in discharge from the current BA session but did not impact future access. Mental health workers were trained to view BA as a learning experience, emphasising that early discharge for contract violations was not considered a failure, and participants' expressions of distress or sadness were acknowledged and validated.

TAU included regular contact with an outpatient psychiatric clinic and access to emergency psychiatric services when necessary. Initially, during the RCT, BA was only available in units capable of psychiatric emergency admissions. Post‐RCT, some clinics established designated BA units where care was exclusively provided by nurses' aides, while others continued to integrate BA services within their emergency psychiatric units.

### 1.4 Measures

The data for this study, presented in more detail below, was obtained from medical records and a 4‐year follow‐up survey.

#### 1.4.1 Medical Records

##### 1.4.1.1 BA Utilisation

Data on BA days, the primary outcome, were collected from medical records at 6‐month intervals for a total of 5 years from the baseline of the RCT. Each interval was designated as a time point and reported separately for the intervention and control groups, as well as for both groups combined. Initially, BA access was restricted to the intervention group and became available to all participants after 12 months, leading to different usage timelines between groups. For the intervention group, T1 represents the 0–6 months period from the baseline of the RCT, while for the control group, gaining access to BA 12 months after baseline, T1 corresponds to the 12–18 month period as measured from baseline. This study uses data from both groups, 48 months from gaining access to BA. The staggered timeline was consistently applied across all eight time points to ensure comparability. The time points, structured in 6‐month intervals, covered a total follow‐up period of 4 years.

##### 1.4.1.2 Days Admitted to Hospital

Data on hospital admissions were extracted from medical records covering the 6‐month period before the 4‐year follow‐up. This included admissions to psychiatric clinics (both voluntary and compulsory care) and hospital stays in other departments due to self‐harm or suicidality. Additional data were collected on coercive measures and hospitalisations in somatic care related to self‐harm and suicidal behaviour (see Lindkvist, Steen Carlsson, et al. 2024).

#### 1.4.2 Four‐Year Follow‐Up Survey

Four years after the conclusion of the RCT, a follow‐up survey was sent to participants who had consented to ongoing data collection. The survey covered gender identity, age, education level, current living conditions, ongoing treatment, self‐harm and suicidal behaviour, functioning, and experiences with BA. Although the survey was carefully developed through extensive discussions within the research team, it was not pre‐tested with potential participants due to practical constraints.

**Non‐Suicidal Self‐Injury (NSSI) and Suicidal Behaviour** were assessed using questions from the Inventory of Statements About Self‐Injury (ISAS; Klonsky and Glenn 2009), measuring whether participants engaged in NSSI during the past week and the frequency of such incidents. Additionally, a yes/no question was included to assess lifetime suicidal behaviour.

**Functioning** was measured using the World Health Organisation Disability Assessment Schedule II (WHODAS II; World Health Organization 2010), a 36‐item self‐report questionnaire assessing six domains: cognition (e.g., concentrating for 10 min; Cronbach's *α* = 0.83), mobility (e.g., getting out of the home; Cronbach's *α* = 0.83), self‐care (e.g., washing the whole body; Cronbach's *α* = 0.56), getting along (e.g., maintaining friendships; Cronbach's *α* = 0.72), life activities (e.g., managing household responsibilities; Cronbach's *α* = 0.95), and participation (e.g., difficulties in living with dignity due to others' attitudes; Cronbach's *α* = 0.84). Items were scored on a 5‐point scale from 1 (no difficulty) to 5 (extreme difficulty/cannot do). Scores were recoded and summed for each domain, ranging from 0 (best) to 100 (worst), using a complex scoring method with an SPSS algorithm provided by the World Health Organisation.

**Participant Experiences and Satisfaction with BA** were assessed in the survey, including questions on whether participants had a current or previous BA contract and whether they had used BA in the past year. In addition to descriptive questions on BA usage, participants with BA experience were asked to rate the quality of the intervention, the extent to which it met their needs, and whether it helped them manage their problems more effectively. These questions were based on a modified version of the Client Satisfaction Questionnaire (CSQ‐8) (Larsen et al. 1979; Attkisson and Zwick 1982). Participants were also asked if they would use BA when needed, recommend it to others, and about their overall satisfaction with the intervention. To complement quantitative responses, open‐text fields allowed participants to elaborate on their experiences.

### 1.5 Qualitative Analysis

The open‐ended responses were analysed using qualitative content analysis (Graneheim and Lundman 2004; Lindgren et al. 2020). Authors RML and RL took the lead in the qualitative analysis, dividing text responses into meaning units, condensing them and then abstracting these condensed meaning units into codes, working with table columns in Microsoft Word. From this point on, we used NVivo 14 to facilitate the overview, sorting and relating of codes in the recontextualisation process. As the depth and detail in text responses varied greatly, from a few words or half sentences to elaborate descriptions, so did the level of analysis of various meaning units. As the data was concrete and the authors wished to remain descriptive regarding influences on BA utilisation, analysis stopped at the categorical level of relatively high abstraction but a low degree of interpretation (Lindgren et al. 2020). All authors were involved in viewing and discussing the analytical levels and tentative qualitative results, reaching agreement that participant responses were credibly represented and that the research aims were appropriately addressed (Graneheim and Lundman 2004). The authors sought to balance appropriate representation of the sometimes quite general descriptions in participant responses, with the aim to describe experiences specific to BA. In the end, when viewed together, the authors believe that the categories generated are indeed specific to BA utilisation.

### 1.6 Statistical Analysis

Statistical analyses were conducted using data from the 63 participants who had complete data over a 4‐year period following their access to BA. We utilised IBM SPSS Statistics 28 and Jamovi 2.2.5 for data processing and analysis. To identify distinct patterns of BA utilisation, we performed a k‐means cluster analysis using the snowCluster package in Jamovi (Seol, 2023). This method was chosen to categorise individuals based on similar BA usage trajectories over time, enabling the identification of distinct utilisation profiles. The analysis incorporated eight time points over a 4‐year period, providing insight into how BA engagement evolved among participants.

To assess differences in sociodemographic and clinical characteristics across BA usage profiles, we employed Fisher's Exact Test for categorical variables, as it is more appropriate for small sample sizes. For continuous variables, we applied one‐way ANOVA, ensuring that the assumptions of approximate normality and homogeneity of variances were met across the groups.

## 2 Results

### 2.1 Descriptive Statistics of BA Utilisation Patterns Over a 4‐Year Period

Table 1 presents descriptive statistics for BA usage across the intervention and control groups, as well as for the total sample, over a 4‐year period. While mean BA usage varied slightly between the groups, no significant differences were observed. The results indicate that BA usage was generally more intensive during the initial phase following implementation but gradually stabilised over time, averaging 3–4 days per 6‐month period. A line graph depicting individual BA frequency across eight time points (T1–T8) is included as Supporting Information (Figure S1).

**TABLE 1:** Means (SD) for BA usage for each 6‐month period following BA implementation.

	T1	T2	T3	T4	T5	T6	T7	T8
Intervention group	9.21 (13.84)	6.49 (10.79)	5.18 (8.53)	5.08 (10.23)	3.51 (7.23)	3.77 (8.12)	5.19 (12.05)	2.97 (6.44)
Control group	5.78 (8.40)	5.17 (7.07)	3.72 (7.00)	4.97 (8.74)	3.79 (8.51)	3.94 (7.23)	3.64 (7.15)	4.38 (8.80)
All	7.75 (11.90)	5.90 (9.30)	4.46 (7.79)	5.03 (9.46)	3.64 (7.80)	3.85 (7.66)	4.40 (9.82)	3.67 (7.68)

*Note:* Since BA became available to the control group only after the first year of implementation and testing, the time frames differ between groups. For the intervention group, T1 represents 0–6 months of use, while for the control group, it represents 12–18 months. The time points summarise BA usage as follows: T1: 0–6 months/12–18 months, T2: 6–12 months/18–24 months, T3: 12–18 months/24–30 months, T4: 18–24 months/30–36 months, T5: 24–30 months/36–42 months, T6: 30–36 months/42–48 months, T7: 36–42 months/48–54 months, and T8: 42–48 months/54–60 months. This structure ensures alignment despite the control group's delayed access to BA.

### 2.2 Classification Based on BA Utilisation Over a 4‐Year Period

A k‐means cluster analysis was conducted on the comprehensive dataset, which comprised eight data points per participant, as presented in Table 1. The objective was to identify distinct BA utilisation profiles reflecting different trajectories of engagement over the 4‐year period. The results revealed that participants could be optimally classified into three utilisation profiles.

**FIGURE 1:**
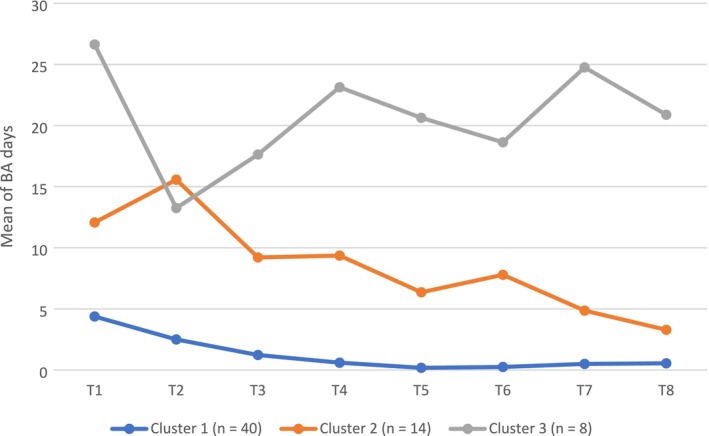
Profiles of BA usage trajectories over a 4‐year period.

As illustrated in Figure 1, the largest group, Cluster 1 (*n* = 40), consisted of individuals who used BA very infrequently, with consistently low usage across all measurement points. Cluster 2 (*n* = 14) included individuals who initially engaged with BA more frequently but exhibited a gradual decline in usage over time. In contrast, Cluster 3 (*n* = 8) represented participants who maintained the highest frequency of BA usage, demonstrating sustained engagement throughout the 4‐year period.

Further analysis indicated that participants from the intervention and control groups were evenly distributed across the three clusters (Cluster 1: 18 [45%] and 22 [55%]; Cluster 2: 8 [57%] and 6 [43%]; Cluster 3: 4 [50%] and 4 [50%], respectively), suggesting that assignment to either group in the RCT study did not significantly influence long‐term usage patterns, *χ* ^*2*^(2) = 0.62, *p* = 0.733.

### 2.3 Descriptive Comparison of Three BA Utilisation Clusters

Table 2 presents descriptive statistics for sociodemographic (e.g., age, education level, living situation) and clinical characteristics (e.g., non‐suicidal self‐injury [NSSI], receipt of psychotherapeutic treatment, hospital admission) across the three BA utilisation clusters. Although no statistically significant group differences were found using Fisher's Exact Test (categorical variables) or one‐way ANOVA (continuous variables), several descriptive trends emerged.

**TABLE 2:** Sociodemographic and clinical characteristics at 4‐year follow‐up following a 1‐year RCT: comparison among three brief admission utilisation Clusters.

Variable	Cluster 1 (*n* = 40)	Cluster 2 (*n* = 14)	Cluster 3 (*n* = 8)
Age, mean (SD)	31.2 (8.8)	38.1 (12.36)	34.6 (9.7)
Woman, No. (%)	30 (75.0%)	12 (85.7%)	6 (75.0%)
Education, No. (%)			
Elementary school or less	4 (11.1%)	1 (9.1%)	1 (14.2%)
High school degree	16 (44.4%)	5 (45.5%)	3 (42.9%)
Bachelor's/Master's degree or higher	14 (38.9%)	4 (36.4%)	3 (42.9%)
Other	2 (5.6%)	1 (9.1%)	0 (0%)
Living alone, No. (%)	21 (52.5%)	4 (36.4%)	3 (37.5%)
Living with partner, No. (%)	14 (35%)	5 (35.7%)	4 (50%)
Accommodation with access to staff during parts of the day, No. (%)	2 (5%)	0 (0%)	0 (0%)
Accommodation with access to staff throughout the day, No. (%)	2 (5%)	0 (0%)	0 (0%)
Child (−ren) at home, No. (%)	6 (15%)	5 (35.7%)	2 (25%)
Received psychotherapeutic treatment at the psychiatric clinic. No. of Yes (%)	14 (35%)	0 (0%)	1 (12.5%)
Received counselling support at the psychiatric clinic. No. of Yes (%)	15 (37.5%)	7 (50%)	4 (50%)
Received help with medication management at the psychiatric clinic. No. of Yes (%)	10 (25%)	2 (14.3%)	5 (62.5%)
Support measures from the municipality/social services. No. of Yes (%)	14 (35%)	5 (35.7%)	5 (62.5%)
NSSI (last week), No. of Yes (%)	1 (2.8%)	4 (36.4%)	3 (42.9%)
NSSI, M/Mdn (SD)	6^a^	9.5/5.5 (11.21)	10.7/5 (13.43)
Suicidal behaviour, lifetime, No. of Yes (%)	31 (86.1%)	10 (90.9%)	6 (87.5%)
M/Mdn (SD) of days admitted to hospital	10.43/0 (24.26)	8.79/0 (21.61)	12.63/0 (22.96)
M/Mdn (SD) of days with compulsory admission to hospital	2.25/0 (9.81)	6.07/0 (21.59)	0.25/0 (0.71)
M/Mdn (SD) of compulsory measures (restraint, forced treatment)	0/0 (0)	0/0 (0)	0/0 (0)
M/Mdn (SD) of visits at an emergency department	1.50/0 (4.66)	1.93/0 (4.25)	1.13/0 (1.89)
WHODAS cognition	30.41 (22.02)	35.00 (22.58)	47.14 (13.50)
WHODAS mobility	20.36 (20.91)	21.02 (28.95)	22.32 (10.74)
WHODAS self‐care	16.67 (19.42)	28.18 (29.26)	35.71 (28.78)
WHODAS getting along	35.24 (28.16)	43.94 (31.42)	34.52 (34.84)
WHODAS domestic responsibilities	33.43 (30.96)	41.82 (40.20)	51.43 (34.36)
WHODAS participation	37.02 (23.65)	31.82 (22.92)	46.53 (22.43)

^a^ Only one person in Cluster 3 reported NSSI, resulting in missing Mdn and SD for this variable.

Participants in Cluster 1 (*n* = 40) were younger (M = 31.2, SD = 8.8) than those in Cluster 2 (*n* = 14; M = 38.1, SD = 12.4) and Cluster 3 (*n* = 8; M = 34.6, SD = 9.7). The majority in all clusters were women (75%–86%), and most had completed at least high school. Living alone was most common in Cluster 1 (52.5%), while Clusters 2 and 3 more often lived with a partner. Staff‐supported housing was rare. Psychotherapeutic treatment was most common in Cluster 1 (35%), while Cluster 3 had the highest use of medication management (62.5%). Counselling support was reported by approximately half of participants in Clusters 2 and 3.

Recent NSSI was uncommon in Cluster 1 (2.8%) but more prevalent in Clusters 2 (36.4%) and 3 (42.9%). Lifetime suicidal behaviour was consistently high across all clusters (≥ 86%).

Functional impairment (WHODAS) was descriptively greater in Cluster 3—particularly in the domains of self‐care, domestic responsibilities, and participation—indicating greater functional difficulties among individuals in this profile. A trend‐level difference in WHODAS self‐care approached significance (*F* (2, 51) = 2.62, *p* = 0.083, partial *η* ^2^ = 0.067). Pairwise comparisons indicated a large difference between Clusters 3 and 1 (Cohen's *d* = 0.91) and a moderate difference between Clusters 3 and 2 (*d* = 0.52), suggesting a possible link between higher BA usage and greater self‐care difficulties.

Table 3 presents responses to questions regarding experiences and perceptions of BA across the three BA usage clusters. Cluster 3 (high BA usage) consistently reported the most positive experiences and highest engagement with BA, whereas Cluster 1 (low BA usage) had the lowest levels of engagement and satisfaction. Cluster 2 (moderate BA usage) displayed an intermediate pattern in both usage and perceived benefit.

**TABLE 3:** Responses to the survey questions on BA.

Variable	Cluster 1 (*n* = 40)	Cluster 2 (*n* = 14)	Cluster 3 (*n* = 8)
Do you have a current contract for BA? Yes/No/Missing response (%)	18 (45%)/18 (45%)/4 (10%)	7 (50%)/4 (28.6%)/3 (21.4%)	6 (75%)/1 (12.5%)/1 (12.5%)
2 Have you used BA in the past year? Yes/No/Missing response (%)	4 (10%)/30 (75%)/6 (15%)	4 (28.6%)/7 (50%)/3 (21.4%)	7 (87.5%)/0/1 (12.5%)
3 How do you assess the quality of BA at your clinic? No. (%)			
Excellent	1 (2.5%)	3 (21.4%)	4 (50%)
Good	1 (2.5%)	1 (7.1%)	3 (37.5%)
Moderate	2 (5%)	0	0
Poor	0	0	0
Missing response	36 (90%)	10 (71.5%)	1 (12.5%)
4 Do you receive the type of care you desire when you are admitted with BA? No. (%)			
Yes, definitely	3 (7.5%)	3 (21.4%)	3 (37.5%)
Yes, for the most part	0	1 (7.1%)	4 (50%)
No, not really	1 (2.5%)	0	0
No, definetly not	0	0	0
Missing response	36 (90%)	10 (71.5%)	1 (12.5%)
5 To what extent does BA meet your needs? No. (%)			
Almost all of my needs	0 (0%)	2 (14.3%)	2 (25%)
Most of my needs have been met	4 (10%)	2 (14.3%)	4 (50%)
Only some of my needs have been met	0	0	1 (12.5%)
None of my needs have been met	0	0	0
Missing response	36 (90%)	10 (71.4%)	1 (12.5%)
6 Has BA helped you to better handle your problems? No. (%)			
Yes, BA has helped a lot	1 (2.5%)	3 (21.4%)	6 (75%)
Yes, BA has helped to some extent	2 (5%)	1 (7.1%)	1 (12.5%)
No, BA hasn't really helped	1 (2.5%)	0	0
No, BA seems to have made it worse	0	0	0
Missing response	36 (90%)	10 (71.4%)	1 (12.5%)
7 Overall, how satisfied are you with BA?			
Very satisfied	1 (2.5%)	3 (21.4%)	5 (62.5%)
Largely satisfied	3 (7.5%)	1 (7.1%)	2 (25%)
Indifferent or somewhat dissatisfied	0	0	0
Completely dissatisfied	0	0	0
Missing response	36 (90%)	10 (71.4%)	1 (12.5%)
8 If you needed help, would you seek BA?			
Yes, definitely	1 (2.5%)	3 (21.4%)	6 (75%)
Yes, I think so	3 (7.5%)	1 (7.1%)	1 (12.5%)
No, I don't think so	0	0	0
No, definitely not	0	0	0
Missing response	36 (90%)	10 (71.4%)	36 (90%)

Participants in Cluster 3 were the most likely to currently hold a BA contract (75%) and to have used BA in the past year (87.5%), compared to 50% and 28.6% in Cluster 2, and 45% and 10% in Cluster 1, respectively. They were also the most likely to rate the quality of BA as excellent (50%), while the majority of participants in Cluster 1 did not provide a response to this question.

Regarding the effectiveness of BA, 75% of Cluster 3 participants reported that BA had helped them significantly, whereas only 21.4% in Cluster 2 and 2.5% in Cluster 1 reported the same. Similarly, 62.5% of Cluster 3 participants expressed being very satisfied with BA, compared to 21.4% in Cluster 2 and 2.5% in Cluster 1. When asked whether they would seek BA again if needed, 75% of Cluster 3 participants responded ‘yes, definitely’, compared to 21.4% in Cluster 2 and only 2.5% in Cluster 1.

The high proportion of missing responses in Cluster 1 indicates that individuals with minimal BA engagement may be less likely to evaluate or reflect on their experiences with BA.

### 2.4 Qualitative Exploration of Participants' Open‐Ended Accounts

Participants in all clusters were represented in the qualitative material, with Cluster 3 participants contributing the most responses, reflecting their higher BA usage profile. The number of participants per cluster in the qualitative analysis was not unproportional considering the differences in cluster sizes in the overall quantitative analysis. Representation of the different psychiatric departments across quotes was similarly proportional.

**FIGURE 2:**
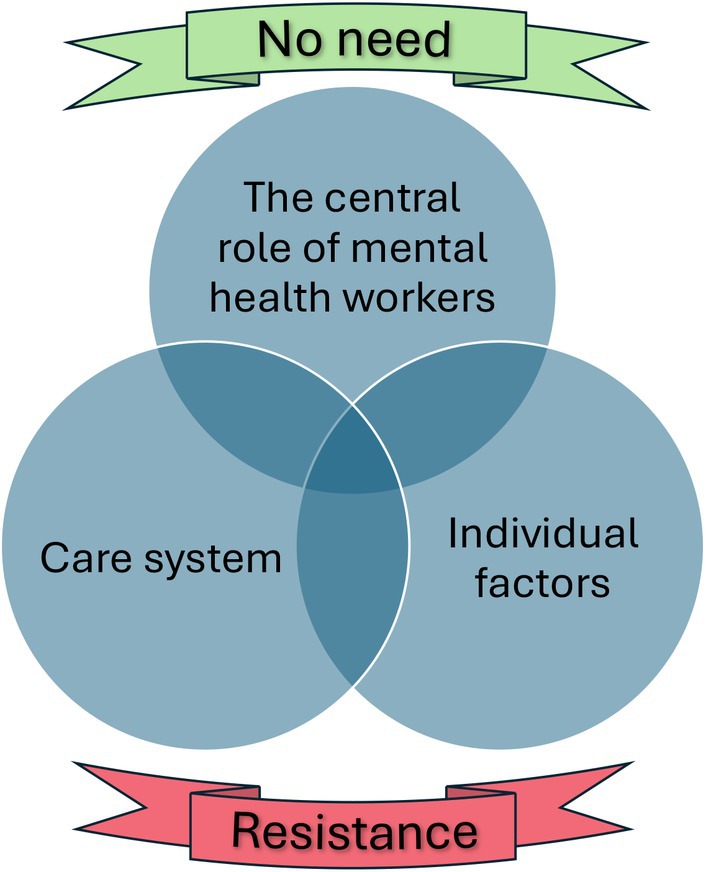
Illustration of the qualitative interpretation of responses on BA utilisation.

In our exploration of participants' free accounts regarding BA utilisation and experiences, we abstracted five categories. Factors related to experiencing *no need* for BA or feeling *resistance* towards BA both contributed to decreased utilisation, though for very different reasons. There were also more multidimensional factors that could make participants more or less prone to use BA, including *individual factors*, *the care system*, and *the central role of mental health workers*, as illustrated in Figure 2. All of these elements interacted in complex ways to influence participants' utilisation of BA.

#### 2.4.1 No Need

Participants described how reasons for not using BA were primarily related to their own needs but also to the needs of others: ‘I'm stable enough with my mental health, so that spot should go to someone else’ (M27, cluster 1.).

Participants described changed circumstances where they were feeling better, good even, and had stopped self‐harming because it no longer fulfilled any purpose. They wrote about no longer fulfilling criteria for any diagnoses and how prior medical or psychiatric treatments had been terminated. As BA contracts had expired, there had been no need for renewal: ‘I haven't needed it [BA**]**! Wohoo!’ (M20, cluster 1.)

Participants shared experiences of no longer needing BA as they had gained access to treatment such as dialectic behavioural therapy or had recovered and found tools of their own to lead a functional life. In some cases, utilisation of any outpatient or inpatient psychiatric care, including BA, had stopped years before follow‐up.

#### 2.4.2 Resistance

Participants also wrote about experiences related to resistance to psychiatric care and how this made them avoid BA despite a felt need for the intervention. Resistance could be related to psychiatric inpatient care in general or specifically to BA. The former could be about the resistance towards being at a clinic, worrying or fearing having to go through an assessment. Participants reported avoiding or postponing BA due to prior care trauma. Suffering from the consequences of prior compulsory admissions could result in avoiding BA altogether or turning to BA too late.

As I had to use [BA] at the [same] department where I was traumatized, I didn't want to admit voluntarily. With BA, you're supposed to use it before it gets that bad. I refused to step foot in the hospital before I was doing really poorly, and then once I got to the hospital I was doing too poorly. (M03, cluster 1)

Resistance to BA could be about resistance to change, sensing that BA was a big project where you had to pack your things, go there, and feel disconnected from normal life. Participants noted feeling resistance but still being able to consider BA during times of high risk of self‐harm. Resistance to BA could be about the struggle to initiate contact, the difficulty getting started with BA, and increased resistance over time when not having used BA for some time, raising doubts: ‘Haven't dared to [use BA] since I haven't done it for a very long time’ (L47, cluster 1.).

Some participants conveyed how awful it was to seek help with BA when feeling bad. Despite trusting the framework, resistance was still present, delaying BA: ‘It still happens that I feel so poorly that I have to wait at home for some days, to have the courage/the ability to come [in for BA]’ (M18, cluster 3.).

#### 2.4.3 Individual Factors

Individual factors were personal circumstances that either supported the individual's process of recovery, that is, contributed to lessening the need for BA, or were described as personal hurdles to using BA.

For instance, basic need fulfilment in terms of for example, an improved home environment was cited by one individual: ‘I've landed such a nice apartment, close to the city center and close to the forest. I'm so happy to live in such a nice place’ (K01, cluster 2.). Others reported positive influences of getting a job, improved self‐esteem, having a greater sense of stability in life, or finding ‘a meaningful everyday life’ (L08, cluster 1). Private relationships were frequently reported to be impactful, such as receiving support from friends and family, or terminating relationships which contributed to personal stress: ‘I used BA to a larger extent before separating. Now I live on my own, hence less stress or urgent need of getting away from home’ (L18, cluster 2.).

One participant explicitly recognised that they ‘should use [BA] more often’ than they did (L32, cluster 3). In terms of personal hurdles, participants referenced feeling worse too quickly so that by the time they recognised a need for help, they felt they were too far gone and ‘not ready for a BA’ (L16, cluster 1), needing regular emergency care instead. Participants also reported feeling guilty when using BA, with regards to other patients or ‘to [my] family, as if I'm opting away from them’ (L32, cluster 3). One participant stated that their needs were ‘exorbitant’, suggesting it would be unreasonable for them to take up space during BA (L40, cluster 3). Another stated that they had decided to avoid psychiatric care altogether, as they were in the process of recovering from and trying to comprehend previous negative experiences of for example, compulsory care.

#### 2.4.4 The Central Role of Mental Health Workers

Participants suggested that their experiences with mental health workers as they called in or were admitted with BA were ‘absolutely essential when you're fragile and have probably waited for too long’, possibly helping to overcome ‘ambivalence’ about seeking care (L54, cluster 2). The same participant also suggested that ‘reasoning about my mental state together with BA workers, can result in me not even needing to seek [BA]’. (L54, cluster 2.) Participants gave several examples of positive experiences with mental health workers, which rendered their admissions with BA more helpful and ultimately encouraged BA usage, as well as examples of negative experiences deterring BA usage.

Participants reported BA‐specific negative experiences as well as negative experiences with workers in psychiatric inpatient care in general, which made them less prone to use BA. Among the more general negative experiences, participants reported perceptions of being wrongfully assessed, wrongfully admitted to compulsory care and losing faith in psychiatry at large. As for BA specifically, one participant shared bad experiences with calling in to seek BA and never hearing back from workers. Another criticised workers on the night shifts, particularly, for seeming ‘unavailable’ and undedicated, ‘snoring so loudly in the day room that I have to close the door to be able to sleep’, not interacting with patients and seemingly influencing other workers to be less dedicated as well, to the effect that ‘patients won't ask for help, and then you could claim that patients are not seeking help’, suggesting that patients were problematized instead of the workers (M18, cluster 3). Others simply felt that the workers on site during BA were unpleasant, stated that they liked some but were annoyed by others, or putting it more diplomatically: ‘some combinations of workers result in me not getting the most of the conversations offered [during BA]’ (L54, cluster 2).

In terms of positive experiences with mental health workers, participants said it was helpful just to get to talk to someone beyond one's personal social network. Talking to mental health workers could be a helpful form of reality‐check for the individual, particularly for participants who ‘often get paranoid’ (K01, cluster 2). Workers were said to ‘always take their time and listen attentively’ (H04, cluster 2), to get to know each individual. One participant explicitly reported positive experiences of getting to talk to the same mental health workers over time during BA. Participants reported that workers encountered them in the same respectful and human manner no matter if they were in good or bad shape during BA, and that they flexibly responded to the individual's needs without questioning them. This was sometimes contrasted to previous experiences with psychiatric inpatient care in which the individual had felt constantly questioned and pulled into a ‘power struggle that constantly arises within institutions, [but] disappears with BA’ (M18, cluster 3).

#### 2.4.5 The Care System

Furthermore, participants reported both positive and negative experiences with the framework and conditions of BA, as well as more general obstacles attributed to the care system and positive experiences of integration of care, which all influenced BA usage to some extent.

Unsurprisingly, several participants were happy to report that psychiatric inpatient care had become more available to them after getting access to BA. One participant touchingly declares, ‘BA has helped me incredibly much in my recovery. It is my *safety net* and I am so grateful that [BA] exists’. (L10, cluster 3, emphasis added.) Several participants shared that having access per se was helpful and reassuring, to the extent that ‘knowing that I have the possibility to use BA has helped me tolerate distressing times’ (L29, cluster 1). In essence, care becoming more available meant that participants felt less of a need to utilise care, which was ‘not to be underestimated!’ (M11, cluster 1.) Participants felt safe having access to BA and felt safe during BA. This was contrasted with the experience of seeking regular psychiatric inpatient care, where not knowing whether one will receive help was described as energy‐demanding, discouraging help‐seeking. Relatedly, the enhanced autonomy and responsibility of the individual was described as helpful, for example, in ‘not needing to convince someone of how sick you are’ (M18, cluster 3) and not having to be assessed or labelled as sick at all. In contrast, one participant described having negotiated an agreement of getting to be assessed professionally upon their own request, as an example of how they had exercised their autonomy in the context of BA. Participants also expressed that it was helpful to seek care preventively, which would then increase their BA utilisation in the short run but decrease other admissions, where ‘before, I could stay admitted for months in compulsory care’ (M13, cluster 3). One participant even suggested that mastering the preventive orientation of BA would, in fact, render the intervention unnecessary in the long run: ‘I've learned to seek help on time. Now I don't need BA anymore’. (K01, cluster 2.) Several participants described the BA framework as helpful ‘when you need to retire and recover’ (M18, cluster 3).

Some downsides to the BA framework were reported, including placing too high demands on the individual to take responsibility for their own medication and make arrangements to get to the psychiatric department, such as packing their bags, planning the trip or contacting someone to watch the dog. Some participants stated that they would have needed an additional day or two of BA, as ‘when I'm using [BA], it feels like I get there, [I barely] have time to land, and then I'm supposed to go home again’ (K08, cluster 1). Some stated that they would have benefited from getting to self‐admit with BA during the evening and night, which was not the case at their unit. Others suggested that BA was simply unhelpful or did not involve proper care: ‘[BA] is mostly a place for storage, where you go to take care of yourself. If you are feeling too poorly to do the whole job yourself, you won't [get] much out of it.’ (M11, cluster 1.)

Participants recognised more general obstacles in the care system, such as when BA was provided in the same department as general psychiatric inpatient care. This was described as a problem in part because of previous negative or even traumatic experiences with care in that same department. The care unit would also become temporarily inaccessible at times due to covid or other factors. Several participants further described a lack of resources making BA practically unavailable, such as shortages of beds and mental health workers in inpatient care, as well as a lack of outpatient contacts meaning that participants ‘had no personal care contact or therapist who might encourage me to seek [BA]’ (M09, cluster 1). On the topic of autonomy, one participant shared having reported certain structural problems they had noticed while utilising BA, though realising their own influence over the care system was limited: ‘It got a bit better once I talked to the old boss, but now it's worse again’. (M18, cluster 3). Worryingly, there also seemed to be a problem concerning the application and integration of BA with regular psychiatric emergency care:

[W]hen you are seeking emergency care, they'll tell you that they can't help you because you have [access to] BA. I can only get help if I have used up all three [BA] admissions. If not, it's just – you've got [access to] BA so you won't get any help from here. I don't feel like I'm getting the help I need when I seek emergency care. (K08, cluster 1.)

This experience of being denied emergency care due to BA and not feeling listened to in their expertise of their own state and needs, led one participant to terminate their BA contract ‘because there were too many arguments about it’ (L16, cluster 1).

On a brighter note, a few participants reported experiences of BA being well integrated with other psychiatric care as well as care that they received in their home. BA workers were reported to occasionally help an individual to seek emergency care. In another case, a participant described that care workers providing so called ‘housing support’, a form of care through home visits focused on daily functioning, had been made aware of BA and would regularly ‘make sure that I'm functioning and stabile. In case of doubt, [they] will advise me to get in contact with BA, though most often I have done so already. On rare occasions, [they've] helped me get to BA’ (L54, cluster 2.).

## 3 Discussion

In the present study, we explored the utilisation of BA over a 4‐year follow‐up period after the RCT using both quantitative and qualitative approaches in a sample of individuals who gained access to BA due to recurrent self‐harm and suicidal ideation.

For both the intervention and control groups, the trajectories of BA usage days over 4 years were similar. Usage was generally more intensive during the initial phase following access to BA but gradually stabilised over time, averaging 3–4 days per 6‐month period. When analysing the data for distinct usage patterns, cluster analysis identified three distinct profiles of BA utilisation, each demonstrating a unique pattern of BA days used over the 4‐year period. Consistent with descriptive statistics for the entire sample, all three clusters showed the highest BA utilisation at the outset, likely reflecting the severity of the condition at initiation and possibly a novelty effect. However, the usage patterns diverged significantly over time. In two clusters, BA usage gradually declined, with Cluster 1, the largest group, showing minimal utilisation during the last follow‐up years. In contrast, Cluster 3, the smallest group, consisting of eight participants, maintained a consistently high level of BA usage throughout the 4‐year period.

No significant differences were found between the clusters on the studied variables. However, an important trend emerged: individuals who consistently utilised BA (Cluster 3) reported greater difficulties in certain areas of functioning, particularly self‐care, as measured by WHODAS. This suggests a potential link between self‐care challenges and long‐term BA engagement, where those experiencing greater impairments in daily functioning maintained higher BA usage over time. These individuals also expressed the highest levels of satisfaction with BA, highlighting benefits such as increased autonomy, not having to fight for or exhaust themselves seeking care, feeling connected with respectful mental health workers, and having their needs taken seriously—all of which may have contributed to their continued use of BA. They also reported receiving support from mental health workers in seeking emergency care when needed. In this sense, BA could be considered a form of self‐care, allowing individuals to actively seek and receive help despite struggles in other areas of daily functioning. Alternatively, it may serve as a gateway to broader care, facilitating access to support when self‐care is challenging. However, some participants in this cluster also acknowledged that they should use BA more frequently than they did. Feelings of guilt and hesitation sometimes prevented them from seeking BA even when they recognised the need, aligning with the quantitative findings on self‐care difficulties.

The overall decline in BA utilisation may be attributed to several factors, with one possible explanation being the alleviation of symptoms associated with BPD. Although this was not examined in the present study, a meta‐analysis by Álvarez‐Tomás et al. (2019) found that 50% to 70% of individuals with BPD achieve long‐term remission, which may contribute to a decreased reliance on BA services over time. Additionally, some individuals may experience help‐seeking barriers and abstain from using BA due to self‐doubt about their right to access it for preventive purposes (Lindkvist, Westling, Liljedahl, and Landgren 2021; Lindkvist, Westling, Eberhard, et al. 2021; Värnå et al. 2025), negative attitudes from clinicians, and a shortage of available beds, often leading to frequent rejections (Helleman et al. 2018). The latter has left relatives feeling betrayed and helpless, further discouraging individuals from seeking help promptly (Lantto et al. 2023; Lindkvist, Eckerström, et al. 2024; Hultsjö, Appelfeldt, et al. 2023). Being denied access to BA due to a shortage of beds or avoiding seeking contact regarding BA due to experiences of unknowledgeable staff or feelings related to fear or guilt has been seen in other recent research, including user experiences of BA (Hultsjö et al. 2025). Similar negative experiences with unavailable care and difficult interactions with mental health workers were reported by participants in the present study. Those in Clusters 1 and 2, when explaining their lack of BA utilisation in the past year, cited a combination of factors, including negative interactions with mental health workers, systemic issues such as staff shortages, difficulties accessing emergency care with BA, and the restrictiveness of the BA framework. Some participants also reported personal resistance, such as reluctance to be admitted or avoidance of specific hospital departments based on past experiences. The care environment has been mentioned in previous research on experiences among users as potentially creating resistance towards using BA (Värnå et al. 2025). Others described individual circumstances, such as improvements in life situations that reduced their need for BA, or no longer perceiving a need for it.

Most of these findings align with previous research (Helleman et al. 2018; Lindkvist, Westling, Liljedahl, and Landgren 2021; Lindkvist, Westling, Eberhard, et al. 2021; Värnå et al. 2025), though this study is the first to confirm that such experiences may persist even 4 years later. The reported difficulty in accessing psychiatric emergency care while holding a BA contract is particularly noteworthy, as this contradicts intended procedures. To the authors' knowledge, this study is the first to report this issue as a reason for certain individuals terminating their BA agreement. Importantly, while some participants experienced barriers to using BA, others found reassurance in simply having a BA contract. Knowing that support was available if needed provided psychological comfort and security, potentially reducing the actual need to use BA. This aligns with findings from previous research, where having access to BA, even when rarely utilised, was described as beneficial in itself (Lindkvist et al. 2019; Lindkvist, Westling, Liljedahl, and Landgren 2021; Lindkvist, Westling, Eberhard, et al. 2021; Lindkvist, Eckerström, et al. 2024; Värnå et al. 2025).

### 3.1 Strengths and Limitations

One of the key strengths of this study is its longitudinal design, with a follow‐up period of four to 5 years after the introduction of the BA intervention, making it the first study in the field of BA with such an extended timeframe. Another notable strength is the use of mixed methods for data collection and analysis, allowing for a richer and more nuanced interpretation of the results.

However, the conclusions of this study are limited by a relatively high dropout rate, which was anticipated given the severity of the sample—a challenge that is common even in studies involving non‐clinical populations (e.g., Gustavson et al. 2012). The modest sample size likely increased the uncertainty of the cluster analyses and reduced the statistical power to detect meaningful differences between clusters across sociodemographic and clinical variables. Given that several hundred participants are typically required for robust person‐oriented approaches—such as cluster analyses or latent profile analyses (e.g., Nylund et al. 2007)—and that most clinical samples with BA data tend to be relatively small, there is a clear need to combine samples from multiple regions in Sweden and/or other Nordic countries to strengthen the robustness of future analyses. Such efforts would not only enable potential validation of the clusters identified in the present study but also allow for more nuanced exploration of sociodemographic and clinical characteristics across different BA utilisation profiles. Furthermore, the qualitative data offered only a partial understanding of participants' experiences, particularly regarding reasons for not using BA, which limits the transferability of findings to low‐ or non‐user populations.

## 4 Conclusions

Study results indicated that BA utilisation declined over time for most participants. However, a small subgroup with persistent self‐care difficulties maintained high engagement throughout the follow‐up period. Despite reporting greater functional impairments, these individuals expressed the highest levels of satisfaction with BA, emphasising its value in providing structured support, autonomy, and a sense of safety. Nonetheless, several barriers to sustained BA use were identified, including self‐doubt, negative clinician attitudes, and systemic challenges such as bed shortages. These obstacles often led to repeated rejections and limited access to emergency psychiatric care while under a BA contract, ultimately prompting some participants to terminate their agreements.

## 5 Relevance to Clinical Practice

This study provides three key insights with important implications for clinical practice:

First, BA emerges as a promising, person‐centred self‐care option that consistently promotes autonomy over time for individuals with severe mental health conditions—particularly those experiencing significant functional impairments and facing barriers to accessing traditional psychiatric services.

Second, a commonly expressed concern among stakeholders—that providing users with unrestricted access to inpatient care might lead to overcrowded wards or excessive healthcare use—appears to be unsupported by the data. Continued use of BA was limited to a small subgroup of participants with the most severe psychiatric symptoms. For this group, BA likely functions as a substitute for other, often more resource‐intensive forms of care, rather than adding to the overall service burden.

Third, successful and sustainable implementation of BA requires ongoing efforts to overcome structural and organisational barriers. These include negative or sceptical clinician attitudes, limited availability of beds, and restricted access to emergency services. Without addressing these systemic challenges, access to BA may remain inequitable and outcomes suboptimal.

## Author Contributions

Daiva Daukantaitė: conceptualisation, data curation, methodology, formal quantitative analysis, visualisation, writing – original draft, writing – review and editing. Rose‐Marie Lindqvist: conceptualisation, qualitative data analysis, visualisation, writing – original draft, writing – review and editing. Reid Lantto: qualitative data analysis, visualisation, writing – original draft, writing – review and editing. Sofie Westling: project administration, funding acquisition, conceptualisation, visualisation, writing – review and editing.

## Conflicts of Interest

The authors declare no conflicts of interest.

## Supporting information


Data S1.

## Data Availability

The data that support the findings of this study are available on request from the corresponding author. The data are not publicly available due to privacy or ethical restrictions.
