# Supplementary material for: Brief Admission by Self‐Referral: A 4‐Year Follow‐Up on Utilisation Patterns and Experiences
Source: Int J Ment Health Nurs. 2025 Jul 9;34(4):e70091. doi: 10.1111/inm.70091 (PMC12241490; doi:10.1111/inm.70091)
Supplement: Supplementary file 1 — Data S1. [file INM-34-0-s001.docx]

**Supplementary Material**

**Figure S1.**

*Individual BA Frequency Across Eight Time Points (T1–T8)*
